# Early Metastatic Relapse in Resected Stage IB KRAS G12D Pancreatic Ductal Adenocarcinoma: Limitations of Anatomical Staging

**DOI:** 10.7759/cureus.109660

**Published:** 2026-05-26

**Authors:** Vaishna Anilkumar

**Affiliations:** 1 Internal Medicine, Medical Council, Dublin, IRL

**Keywords:** early-stage cancer, kras mutation, metastasis, pancreatic adenocarcinoma, pancreatic cancer, pancreatic tail tumor, perineural invasion, vascular invasion

## Abstract

Pancreatic ductal adenocarcinoma (PDAC) is associated with poor survival, although patients with localized resectable disease are generally expected to achieve improved outcomes following surgery and adjuvant chemotherapy. Increasing evidence, however, suggests that tumor biology may outweigh anatomical staging in determining prognosis. This case report describes the case of a 36-year-old male with low-risk factors diagnosed with stage IB pancreatic tail adenocarcinoma following evaluation for persistent dyspeptic symptoms. The patient underwent distal pancreatectomy with splenectomy and achieved a margin-negative, node-negative resection. Histopathology demonstrated lymphovascular and perineural invasion, while delayed molecular profiling identified a KRAS G12D mutation. Despite apparently favorable pathological staging and adjuvant FOLFIRINOX chemotherapy, the patient developed early biochemical progression with rapidly rising carbohydrate antigen 19-9 (CA 19-9) levels, followed by widespread metastatic dissemination involving the liver, lung, spine, skeletal muscle, and multiple visceral sites. Notably, disease progression occurred despite a transient biochemical response to second-line chemotherapy, highlighting discordance between tumor marker kinetics and true disease burden. The patient died 13 months after diagnosis. This case highlights the limitations of anatomical staging in PDAC and emphasizes the prognostic importance of tumor biology, including KRAS mutation status, lymphovascular invasion, and perineural invasion. It also demonstrates the potential limitations of CA 19-9 as a solitary marker of treatment response in biologically aggressive disease.

## Introduction

Pancreatic ductal adenocarcinoma (PDAC) remains one of the most lethal solid malignancies, with a five-year survival rate below 10% [1,2]. Although early-stage resectable disease is generally associated with improved outcomes, increasing evidence suggests that tumor biology may be more prognostically significant than anatomical staging alone [3,4].

Histopathological features such as lymphovascular invasion and perineural invasion, together with molecular alterations including KRAS mutations, are increasingly recognized as markers of aggressive disease behavior [5,6]. This case reports a rapidly progressive, stage IB KRAS G12D-mutated PDAC, demonstrating early metastatic relapse despite curative-intent surgery and standard adjuvant chemotherapy.

## Case presentation

**Figure 1:**
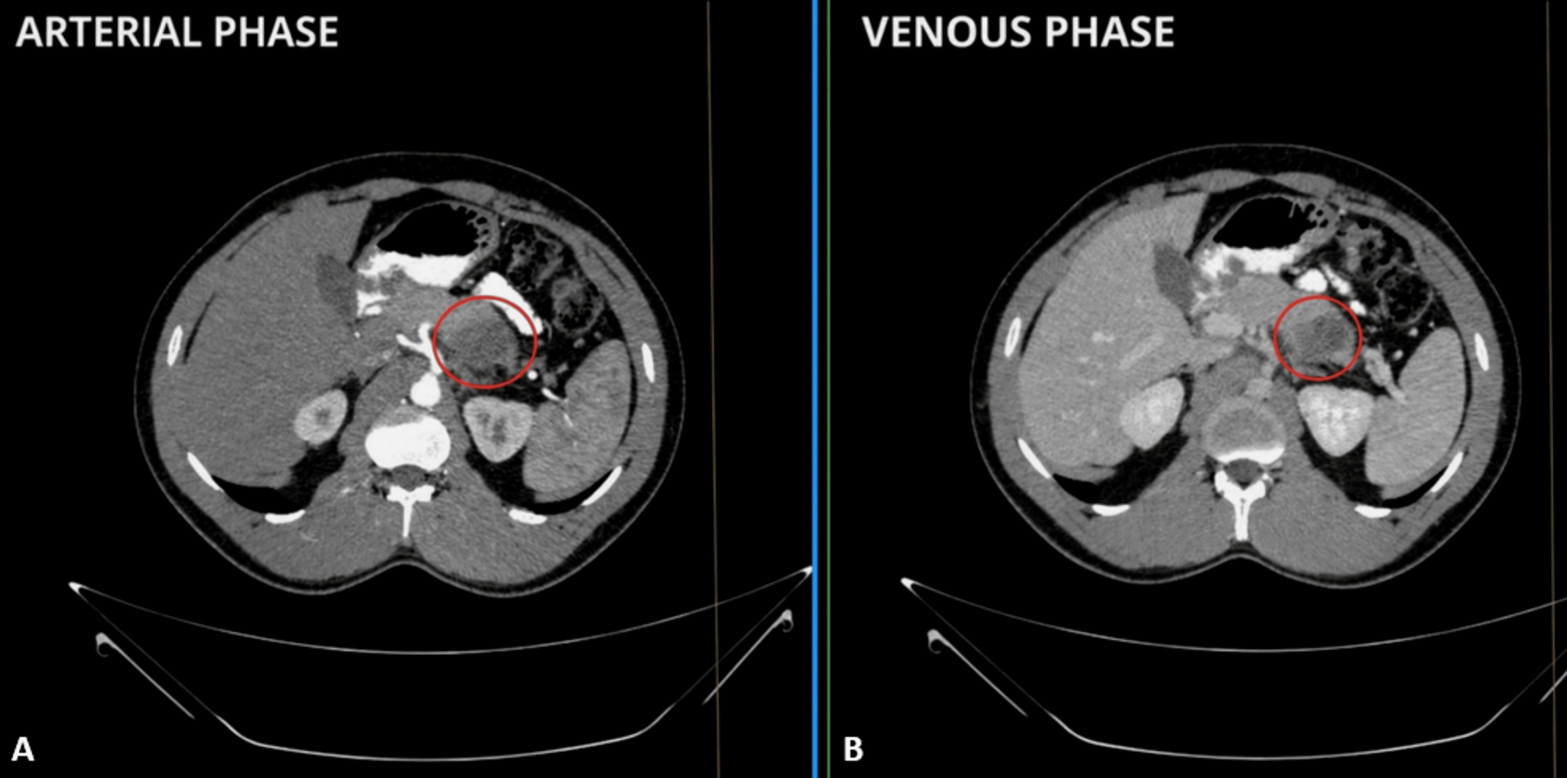
Arterial (A) and venous (B) phase contrast-enhanced CT images demonstrating a hypoattenuating lesion in the tail of the pancreas (circled in red) The lesion is better characterized on venous phase imaging.

A 36-year-old physically active male with no significant comorbidities or major traditional risk factors presented with a six-month history of persistent epigastric discomfort and postprandial dyspeptic symptoms. Initial abdominal ultrasonography and plain radiography were unremarkable, and symptoms failed to improve despite proton pump inhibitor therapy. Owing to persistent symptoms, further evaluation with contrast-enhanced CT was performed, which identified a mass in the pancreatic tail (Figure 1A, 1B). Serum carbohydrate antigen 19-9 (CA 19-9) was elevated at 185 U/mL.

Following multidisciplinary team discussion, the patient underwent distal pancreatectomy with splenectomy and left adrenalectomy. Histopathological examination demonstrated PDAC, characterized by poorly formed glands within a desmoplastic stroma with focal mucin production. The tumor measured 3.5 cm and was confined to the pancreatic tail. Surgical margins were negative, and all 21 examined lymph nodes were free of metastatic involvement, consistent with stage IB disease (pT2N0M0). However, both lymphovascular invasion and perineural invasion were identified [7].

Postoperatively, serum CA 19-9 decreased to 20 U/mL, and adjuvant modified FOLFIRINOX chemotherapy was initiated with curative intent. Despite treatment, CA 19-9 levels increased to greater than 200 U/mL within two cycles, raising concern for early biochemical progression and possible chemoresistance. The patient subsequently developed right-sided back pain, and imaging demonstrated a solitary hepatic metastasis, which was treated with stereotactic body radiotherapy. Second-line systemic therapy with gemcitabine and nab-paclitaxel was subsequently commenced.

Throughout the disease course, the patient persistently complained of diffuse upper back pain; however, repeated clinical evaluations and diagnostic investigations failed to identify a definitive etiology. A PET scan performed approximately two months after initiation of second-line chemotherapy demonstrated pulmonary metastases. Notably, serum CA 19-9 levels declined significantly from values in the 900 U/mL range to approximately 170 U/mL. This biochemical response appeared discordant with the imaging findings, and treatment with the same chemotherapy regimen was continued.

The patient later developed pain localized to the left pubic ramus. CT imaging demonstrated a pathological fracture involving the left pubic ramus. Additional imaging obtained prior to planned palliative radiotherapy revealed extensive metastatic dissemination involving the liver, lungs, spine, skeletal muscle, and multiple visceral sites. This widespread progression occurred within approximately three months of the PET scan that had previously demonstrated only pulmonary metastatic disease.

Delayed molecular profiling subsequently identified a KRAS G12D mutation. By the time molecular results became available, the extensive metastatic burden and rapid clinical deterioration precluded enrollment in clinical trials or consideration of targeted investigational therapies.

The patient experienced rapid functional decline and died approximately 13 months after the initial diagnosis.

## Discussion

This case demonstrates a striking discordance between favorable anatomical staging and highly aggressive clinical behavior in PDAC. Despite stage IB disease with negative lymph nodes and clear surgical margins, the patient developed rapid systemic metastatic progression shortly after curative-intent resection, suggesting occult micrometastatic dissemination at diagnosis.

The presence of perineural and lymphovascular invasion [5] and the KRAS G12D mutation subtype strongly supports a biologically aggressive tumor phenotype [7]. Increasing evidence suggests that tumor biology may provide greater prognostic value than conventional TNM staging alone, particularly in patients with early postoperative relapse and treatment resistance. Similar reports in the literature have described patients with apparently localized PDAC who experienced rapid metastatic recurrence despite favorable pathological staging, highlighting the growing recognition that molecular and histopathological characteristics may better predict disease trajectory than anatomical staging alone.

An important feature of this case was the discordance between the CA 19-9 response and true disease progression. Although tumor marker levels transiently improved during second-line chemotherapy, the patient simultaneously developed extensive metastatic disease. This highlights a major limitation of relying solely on CA 19-9 kinetics as a surrogate marker of therapeutic response in aggressive PDAC and emphasizes the importance of early reassessment when new symptoms arise despite apparent biochemical improvement. Previous studies have similarly demonstrated that CA 19-9 responses may not consistently correlate with radiological or clinical outcomes in biologically aggressive PDAC, particularly in the setting of rapidly evolving metastatic disease.

The development of skeletal muscle metastases was particularly notable, as this represents an uncommon site of spread in PDAC and is generally associated with advanced, aggressive disease [8]. Skeletal muscle metastases from pancreatic adenocarcinoma are rarely reported in the literature and are typically associated with widespread dissemination and poor prognosis. Progressive musculoskeletal pain in this patient preceded recognition of extensive dissemination, emphasizing the need for continued clinical suspicion during treatment.

Overall, this case highlights the limitations of anatomical staging in PDAC and underscores the importance of integrating molecular and histopathological features into prognostic assessment. Earlier identification of biologically aggressive disease may improve risk stratification and guide future personalized therapeutic approaches. Furthermore, the unusual metastatic pattern and rapid postoperative progression observed in this patient contribute to the growing body of evidence demonstrating the heterogeneity and unpredictability of PDAC behavior, even in apparently early-stage disease.

## Conclusions

This case illustrates how early-stage PDAC may behave as a biologically advanced disease despite favorable anatomical staging. Rapid postoperative metastatic progression in the presence of lymphovascular invasion, perineural invasion, and KRAS G12D mutation highlights the prognostic significance of tumor biology beyond conventional TNM classification.

A key clinical takeaway from this case is that biochemical improvement alone may not reliably reflect therapeutic response in aggressive PDAC. Persistent or evolving symptoms should prompt early reassessment even when CA 19-9 levels appear favorable. Although limited by the nature of a single case report, this case’s findings underscore the potential value of incorporating molecular characteristics alongside conventional clinicopathological factors in future prognostic research and personalized treatment strategies in PDAC.
